# Effect of Rotary Swaging on the Structure, Mechanical Characteristics and Aging Behavior of Cu-0.5%Cr-0.08%Zr Alloy

**DOI:** 10.3390/ma16010105

**Published:** 2022-12-22

**Authors:** Natalia Martynenko, Olga Rybalchenko, Anna Bodyakova, Dmitriy Prosvirnin, Georgy Rybalchenko, Mikhail Morozov, Vladimir Yusupov, Sergey Dobatkin

**Affiliations:** 1 A.A. Baikov Institute of Metallurgy and Materials Science of the Russian Academy of Sciences, Leninsky Prospect, 49, 119334 Moscow, Russia; 2 Laboratory of Mechanical Properties of Nanoscale Materials and Superalloys, Belgorod National Research University, Pobeda Street, 85, 308015 Belgorod, Russia; 3 P.N. Lebedev Physical Institute of the Russian Academy of Sciences, Leninsky Prospect, 53, 119334 Moscow, Russia; 4 Department of Metal Science and Physics of Strength, National University of Science and Technology “MISIS”, Leninsky Prospect, 4, 119049 Moscow, Russia

**Keywords:** copper alloys, rotary swaging, aging, mechanical properties, electrical conductivity, fatigue strength

## Abstract

A study of the effect of rotary swaging (RS) on the microstructure and properties of the pre-extruded and pre-quenched Cu-0.5%Cr-0.08%Zr alloy was performed. RS leads to the formation of an ultrafine-grained (UFG) microstructure. UFG structure formation caused by RS increases the ultimate tensile strength (UTS) up to 443 ± 5 MPa and 597 ± 9 MPa for pre-quenched and pre-extruded alloys, respectively. Additionally, the reduction in ductility occurs after RS. It should be noted that UTS is increased for a pre-quenched alloy, while the strength of a pre-extruded alloy is dropped. The growth of UTS for the pre-quenched alloy is associated with the precipitation of fine Cr particles, whereas the recovery processes in the pre-extruded alloy induce the reduction in its UTS. An additional advantage of RS is an increase in the fatigue limit of the pre-quenched alloy up to 265 MPa, and of the pre-extruded alloy up to 345 MPa. The combination of extrusion and RS allows for the increase of the UTS of the Cu-0.5%Cr-0.08%Zr alloy up to 597 ± 9 MPa, while the levels of ductility and electrical conductivity are 10.9 ± 0.9% and 82.0 ± 1.7% IACS, respectively.

## 1. Introduction

The Cu-Cr-Zr alloys are excellent materials for the electrical industry due to their high thermal and electrical conductivity [1,2,3,4]. However, electrical products, such as resistance welding electrodes, often operate under high loads (including cyclic loads) at elevated temperatures. Therefore, materials for such products should have improved mechanical properties and high wear resistance. An increase in the strength of electrical copper alloys without losing their electrical conductivity is a priority task. Low-alloyed Cu-Cr-Zr alloys are typical precipitation-hardened materials. A good combination of strength and electrical conductivity can be achieved due to the decomposition of a supersaturated solid solution (SSSS) and the precipitation of fine particles [5,6,7,8] of the chromium phase and zirconium phase [9]. However, the strength achieved by precipitation hardening is often insufficient for the industrial application of Cu-Cr-Zr alloys. In this regard, there is a need for additional hardening of Cu-Cr-Zr alloys. Severe plastic deformation (SPD) with subsequent aging is a promising treatment for increasing the strength and electrical conductivity of copper alloys [9,10,11,12,13,14,15,16]. This processing makes it possible to obtain grain refinement (up to an ultrafine-grained (UFG) structure) caused by SPD and precipitation during aging, which will significantly increase the strength characteristics, both under static and cyclic loads. In this case, the decomposition of SSSS and the partial relaxation of stresses during heating make it possible to significantly increase the electrical conductivity of copper alloys. Despite these advantages, SPD methods are still poorly implemented in the industry due to the complexity of the process and its high cost. Therefore, the search for industrial deformation methods that make it possible to obtain a stable and uniform UFG structure in metals and alloys is an urgent task. 

Rotary swaging (RS) is one of the industrial ways to solve the problem of operational properties improving. It is known that RS can effectively refine the microstructure of steels [17,18], aluminum [19,20], magnesium [21,22], titanium [23,24] and other alloys. The main advantages of RS are relatively low cost, ease of use and the ability of integration into the technological chain at different stages of thermomechanical processing. This makes it possible to improve the properties of materials by adjusting the structural-phase state of the alloy. Thus, a high level of strength and electrical conductivity was achieved in a low-alloyed Cu-0.3%Cr-0.1%Zr-0.05Mg alloy by the rotary swaging method built into the chain of the two-step aging treatment [25]. It should also be noted that the microstructure refinement by RS is aimed not only at increasing the strength characteristics of the alloy, but also at increasing its wear resistance. Shan’gina et al. showed in [26] that the formation of a UFG structure in the Cu-0.7%Cr-0.9%Hf alloy makes it possible to double the wear resistance of resistance welding electrodes compared to the initial state of the alloy. The RS processing of the Cu-Cr-Zr alloy makes it possible to obtain a UFG structure, which provides a higher level of ultimate strength with increased values of electrical conductivity, wear resistance and thermal stability. Later on, this set of properties can be effectively used to create high-strength and wear-resistant electrical products with high electrical and thermal conductivity and high resistance to softening, for example, in applications such as electronic terminals, connectors and resistance welding electrodes.

The possibility of improving the operational characteristics of the Cu-0.5%Cr-0.08%Zr alloy in two initial states (after quenching and after extrusion) by the RS with subsequent aging was studied in this work. It is expected that the RS of the low-alloyed Cu-Cr-Zr alloy would significantly refine the microstructure, and the aging would make it possible to obtain a high-strength state with a high level of electrical conductivity. The Cu-0.5%Cr-0.08%Zr alloy belongs to the type of precipitation-hardened alloys, that is, its strength can be increased due to the precipitation of small particles of the second phase from a supersaturated solid solution. These phase particles effectively prevent the slip of dislocations, resulting in strengthening. At the same time, the positive effect of SSSS decomposition is not only an increase in strength characteristics but also an improvement in one of the main parameters of the studied alloy—its electrical conductivity. The precipitation of fine particles leads to a decrease in the concentration of the alloying element in the SSSS, which reduces the degree of distortion of the matrix crystal lattice and increases its electrical conductivity. However, deformation can significantly affect the processes of SSSS decomposition by accelerating it or shifting it to lower temperatures. It was previously shown that high-pressure torsion (HPT) accelerates the SSSS decomposition of the low-alloyed bronzes due to the high density of crystal lattice defects [27]. Therefore, the study of the aging processes that occur during heating, as well as the effect of deformation on these processes, is an important stage in the study of low-alloy copper alloys.

## 2. Materials and Methods

**Figure 1:**
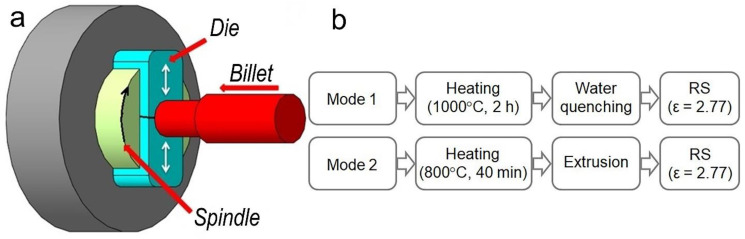
Schematics of the RS process (**a**) and technological modes of alloy processing (**b**).

The Cu-0.5%Cr-0.08%Zr alloy was the studied material in this work. Cu-5%Cr and Cu-10%Zr master alloys, which were prepared from high-purity metals Cu (99.96%), Cr (99.95%) and Zr (99.8%) (hereinafter, wt.%) were melted for the preparation of low-alloyed zirconium bronze. The master alloys were melted in an arc vacuum furnace in an atmosphere of purified argon on a water-cooled copper tray using a non-consumable tungsten electrode. The Cu-Cr-Zr alloy was melted in a vacuum induction furnace in an argon atmosphere in a corundum crucible. The casting was also performed in an argon atmosphere into a graphite mold with a diameter of 50 mm and a height of 120 mm. The alloy was additionally heated in a furnace at 800 °C (~40 min) before extrusion, while the pressing container was heated up to 400 °C. The final billet with a diameter of 25 mm was turned to a diameter of 20 mm, required for subsequent deformation processing. The content of chromium and zirconium in the prepared alloy was 0.5 and 0.08%, respectively, according to chemical analysis. The billets were annealed after extrusion at 1000 °C for 2 h with subsequent water quenching. Rotary swaging was carried out on a rotary swaging machine RKM 2129.02 (maximum force 8 kN) at room temperature (for details see [21]). RS was carried out for the alloy in two initial states: after extrusion at 800 °C, and after quenching at 1000 °C (2 h, water cooling). Total accumulated deformation degree (ε = ln(A_0_/A_f_), where A_0_ and A_f_ are the initial and the final cross-section areas of the billets, respectively) was 2.77. The scheme of the RS process and the technological modes of processing the alloy are shown in Figure 1.

The microstructure of the alloy in the initial states was studied using an Axio Observer D1m Carl Zeiss optical microscope (longitudinal section for extruded samples). The fine structure of the alloy after RS was studied by transmission electron microscopy (TEM) using a JEOL JEM 2100 microscope (Jeol, Tokyo, Japan) with an operating voltage of 200 kV. The samples for microstructural studies were prepared by electrochemical polishing at a temperature of −20 °C in an electrolyte containing 25% HNO_3_ and 75% CH_3_OH at a voltage of 10 V. The dislocation density was calculated by quantifying the number of individual dislocation outcroppings on the foil surface from at least five TEM images. The volume fraction of particles was determined as the average volume of visually distinguishable particles divided by the area of the foil and multiplied by its average thickness (70 nm). The average size of the structural components was estimated by the method of random sections using the Image ExpertPro 3 program.

The study of thermal stability (time = const) and aging kinetics (temperature = const) was carried out by studying the dependence of the microhardness and electrical conductivity of the alloy on heating temperature and time, respectively. At the same time, microhardness measurements were carried out using a 402MVD Instron Wolpert Wilson Instruments automatic hardness tester (Wilson Instruments, Norwood, MA, USA) for determining Vickers microhardness with a load of 100 g and an exposure time of 10 s. The electrical resistance was measured using a BSZ-010-2 microohmmeter (JSC "NIIEMP", Penza, Russia) on flat samples with a width of 4.5 mm and a thickness of 1 mm. The obtained electrical resistivity values were converted to electrical conductivity and given as a percentage of the annealed copper conductivity value in accordance with the International Annealed Copper Standard (%IACS).

The uniaxial tensile properties were evaluated using an Instron 3382 testing machine (Instron, High Wycombe, UK) with an extension rate of 1 mm/min on flat samples with a gauge cross-section of 2 mm × 1 mm and a length of 5.75 mm. Flat specimens with a working section of 1 mm × 1 mm and a working length of 5.75 mm were used for fatigue tests. The tests were carried out according to the scheme of cyclic stretching at a cycle frequency of 30 Hz and a cycle asymmetry coefficient R = 0.1. The tests were performed on the basis of 10^7^ load cycles at room temperature on an Instron Electropuls E3000 servo-hydraulic machine (Instron, High Wycombe, UK) equipped with Instron Wave Matrix software, with a total error of no more than 0.25% of the measured value. The study of the fracture surfaces of fatigue failure was carried out using a scanning electron microscope (SEM) JSM-7001F (Jeol, Tokyo, Japan).

## 3. Results

**Figure 2:**
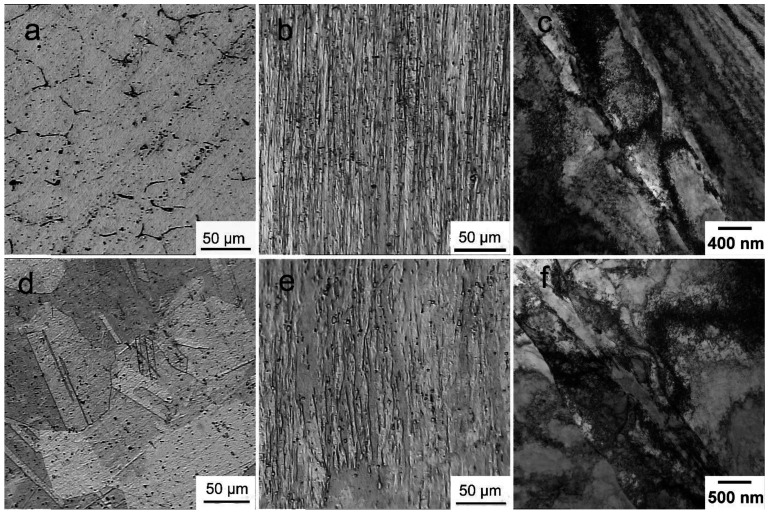
Microstructure of the Cu-0.5%Cr-0.08%Zr alloy in various states: (**a**) extrusion; (**b**,**c**) extrusion + RS; (**d**) quenching; (**e**,**f**) extrusion + quenching + RS.

Figure 2 shows the results of studying the microstructure of the Cu-0.5%Cr-0.08%Zr alloy in various states. The structure of the extruded alloy consists of copper-based solid solution grains with an average size of 44.5 ± 2.4 µm. A fine network of excess phases, remaining from the cast state, is observed at the boundaries of these grains (Figure 2a). The structure of the alloy changes significantly after quenching: grain recrystallization occurs, the network of excess phases is completely dissolved, and annealing twins appear (Figure 2d). The average grain size of the quenched alloy is 161.1 ± 13.3 µm, while the average width of the annealing twins is 6.0 ± 0.5 µm.

Rotary swaging of the Cu-0.5%Cr-0.08%Zr alloy leads to a significant microstructure refinement, both for the initially extruded and initially quenched states. The formation of an elongated shape along the swaging direction structure with a width of elongated grains of ~5–10 µm is observed for both states of the alloy (Figure 2b,e). In both cases, the formation of a grain–subgrain structure is observed inside the elongated grains, characterized by a high density of dislocations, which is 4 × 10^14^ m^−2^ and 5–6 × 10^14^ m^−2^ for the initially quenched and extruded alloy, respectively. The boundaries of the subgrains are formed by wide dislocation walls. The structure contains both elongated grains and shear bands and almost equiaxed subgrains. The formation of structural elements with a size of ~300–400 nm and shear bands with a width of 500–600 nm was revealed in the initially quenched alloy after RS (Figure 2f). The formation of subgrains with a size of ~200 nm and shear bands with a width of 300–400 nm was observed in the case of an alloy swaged after extrusion (Figure 2c). 

**Figure 3:**
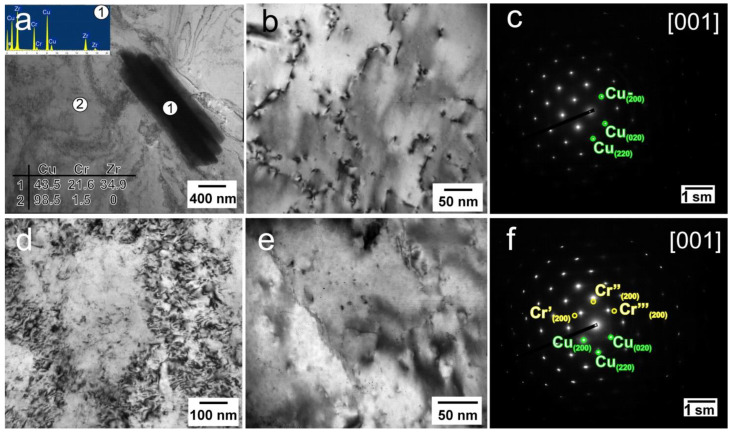
Fine structure of the Cu-0.5%Cr-0.08%Zr alloy in various states ((**a**) extrusion + RS; (**b**) extrusion + quenching + RS; (**c**) SAED pattern with [001] zone axis for alloy after extrusion + quenching + RS; (**d**,**e**) extrusion + RS, (**f**) SAED pattern with [001] zone axis for alloy after extrusion + RS).

Regardless of the initial state, large needle-like particles with a length of more than 1 µm and a width of 300–500 nm are observed (Figure 3a). The particles are rich in Cr and Zr and have an identical chemical composition with the Heusler phase Cu_2_CrZr found in [28]. It should be noted that the diffraction reflections of the {200}_Cu_ and {220}_Cu_ planes are streaked along the <110>_Cu_ directions in the diffraction patterns in the alloy after quenching and RS (Figure 3c), which indicates large internal distortions of the copper matrix [29]. Coherent bcc Cr particles with a size of about 3–4 nm were detected in the extruded alloy, which have a coffee-bean contrast or Moiré contrast (Figure 3d). In the diffraction patterns from particles, there are reflections of the (200)_Cr_ plane (Figure 3e), which are rotated by 0°, 60° and −60° relative to the <110>_Cu_ directions. This corresponds to the Nishiyama–Wasserman orientation relationship for Cr particles precipitated in the early stages of the decomposition of a supersaturated solid solution [29].

**Figure 4:**
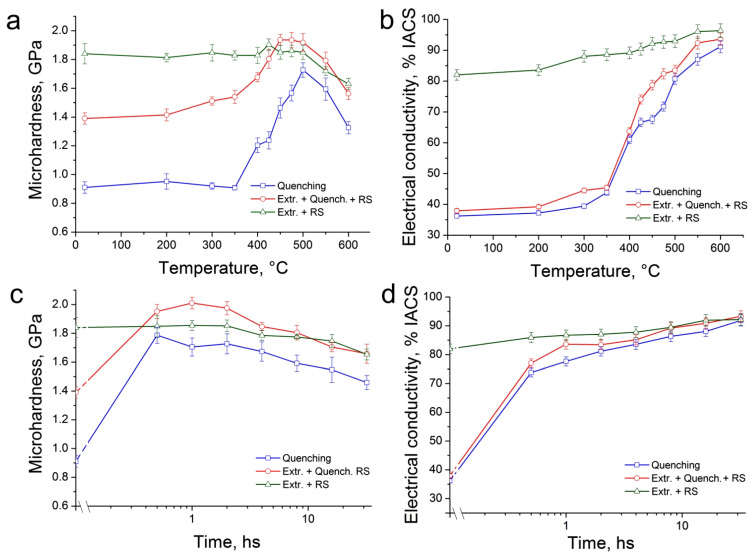
Dependence of microhardness (**a**,**c**) and electrical conductivity (**b**,**d**) of Cu-0.5%Cr-0.08%Zr alloy in various states on temperature (**a**,**b**) and time (**c**,**d**) of heating.

Figure 4 shows the results of studying the aging processes in the Cu-0.5%Cr-0.08%Zr alloy before and after rotary swaging by studying the dependences of microhardness and electrical conductivity on temperature (Figure 4a,b) and time (Figure 4c,d) heating. Thus, the initial value of the microhardness for the alloy in the quenched state is 0.91 ± 0.07 GPa. The RS of the quenched alloy leads to an increase in the microhardness up to 1.39 ± 0.04 GPa due to the refinement of the microstructure. At the same time, the microhardness of the alloy reaches 1.84 ± 0.07 GPa after combined treatment (extrusion + RS). The study of the microhardness behavior of the quenched alloy during aging showed that its values do not change in the temperature range from 20 to 350 °C. Heating the alloy to a temperature above 350 °C leads to a sharp increase in microhardness, a peak of which is observed at 500 °C (1.73 ± 0.05 GPa). Further heating above 500 °C leads to a gradual softening of the alloy. In the case of an alloy swaged after quenching, the microhardness values begin to gradually increase already after heating above 200 °C. It reaches a peak at a temperature of 450 °C (1.94 ± 0.05 GPa) and practically does not change up to 500 °C, after which it sharply decreases. The heating in the temperature range of 20–500 °C does not lead to a significant change in the microhardness of the initially extruded alloy after RS (Figure 4a). Further heating of the alloy above 500 °C leads to gradual softening. As mentioned above, the growth of microhardness is due to the release of fine particles, which prevent free dislocation slip. In this case, the decomposition of SSSS in copper alloys is usually accompanied by an increase in their electrical conductivity. Therefore, the second step in the investigation of aging processes is to study the dependence of the values of the electrical conductivity of the alloy on the heating temperature (Figure 4b). The study of this dependence confirms the hypothesis about the decay of SSSS. Therefore, a slight change in the values of electrical conductivity is noted during heating up to 350 °C for the alloy quenched and swaged after quenching. Heating above 350 °C leads to a sharp increase in electrical conductivity. It is interesting to note that a slight increase in electrical conductivity is also observed after heating in the temperature range of 200–600 °C for the alloy subjected to combined processing (extrusion + RS). A decrease in the density of defects in the crystal lattice during heating, caused by the processes of recovery, can be the reason for this increase in the electrical conductivity of the alloy.

Based on the obtained data, in the first step, the temperatures of isothermal annealing of the alloy are selected, and then in the second step, the duration of aging is chosen based on the annealing temperature. The temperature of isothermal annealing of the alloy in all microstructural states is 500 °C. For this temperature, the optimal combination of microhardness and electrical conductivity was found.

The dependences of microhardness and electrical conductivity on aging time are shown in Figure 4c,d. The study showed that a significant increase in microhardness is observed already after half an hour of heating at 500 °C for the alloy quenched and RS-treated after quenching. In the case of a quenched alloy, a further increase in the heating time to 2 hours leads to a small (within the error) decrease in microhardness. A further increase in heating time leads to a gradual decrease in microhardness. The microhardness peak is observed after heating for 1 hour and changes slightly after 2 hours of heating for the initially quenched deformed alloy. Further heating leads to a sharp softening of the alloy. In this case, heating the alloy after extrusion and RS does not lead to a change in its microhardness after 2 hours of aging. Further annealing in the range of 2–32 hours leads to a slow reduction in microhardness. At the same time, an increase in electrical conductivity is observed for the alloy in all microstructural states (Figure 4d). However, the increase in electrical conductivity is most pronounced for a quenched alloy (quenched alloy and alloy after mode 1 (Figure 1b)), since in this case it is associated not only with the annihilation of defects in the crystal structure but, first of all, with the decomposition of SSSS. In the case of an alloy swaged after extrusion, the increase in electrical conductivity is due only to the annihilation of defects in the crystal structure. Based on the results of the study of the aging kinetics, the aging parameters of the alloys were selected, of which the optimal combinations of microhardness and electrical conductivity were obtained. Heating at 500 °C for 2 hours was chosen as the optimal aging parameters for all microstructural states of the alloy, since at this temperature, the optimal combination of the main parameters is achieved. These aging parameters provide a sufficiently high level of microhardness and electrical conductivity of the alloy. The specific electrical conductivities after aging at 500 °C for 2 hours were 81.2 ± 1.6, 83.4 ± 1.8 and 87.0 ± 1.8% IACS for the alloy after quenching, quenching + RS as well as extrusion + RS, respectively.

**Figure 5:**
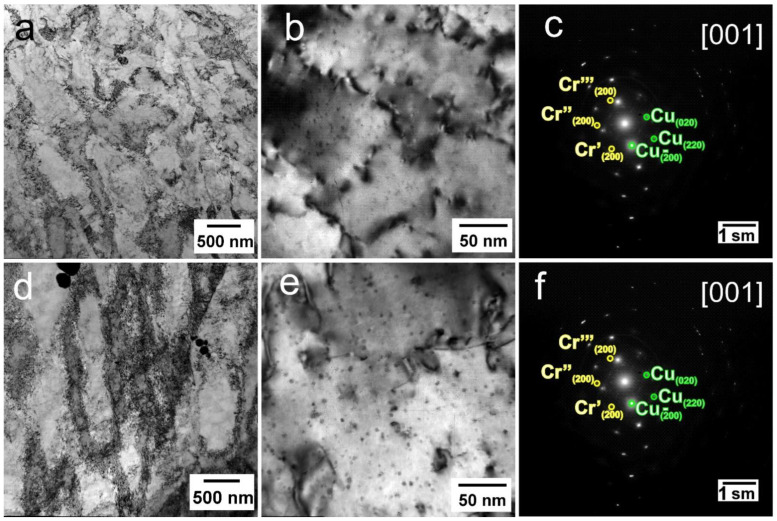
Microstructure of the Cu-0.5%Cr-0.08%Zr alloy after RS and subsequent aging: (**a**,**b**) extrusion + quenching + RS + aging at 500 °C for 2 h and (**c**) the diffraction pattern from the image obtained; (**d**,**e**) extrusion + RS + aging at 500 °C for 2 h and (**f**) the diffraction pattern from the image obtained.

The effect of aging on the microstructure of the alloy after RS is shown in Figure 5. 

A grain–subgrain microstructure is mostly observed in the Cu-0.5%Cr-0.08%Zr alloy after quenching + RS + aging at 500 °C (Figure 5a). The dislocation density slightly decreases during annealing to 4–5 × 10^14^ m^−2^, and the subgrain size increases to 400–500 nm. In addition, a precipitation of dispersed particles with a size of about 3–5 nm is observed in the structure (Figure 5b). According to the diffraction analysis, these are bcc Cr particles (Figure 5c). The structure of the pre-extruded swaged Cu-0.5%Cr-0.08%Zr alloy after aging is shown in Figure 5d. In this case, a decrease in the dislocation density to 3–4 × 10^14^ m^−2^ is observed after aging. The subgrain structure changes slightly after heating. Dislocation cells with an average size of 400–600 nm and subgrains with a size of ~300 nm are discovered. At the same time, dispersed Cr particles are observed in the structure after extrusion + RS + aging, as in the case of the alloy after quenching + RS + aging (Figure 5e,f). Aging contributed to a slight growth up to 4–6 nm of chromium particles formed during extrusion. It is possible that fine particles enriched in Zr, which were observed in [9,25,30], are also present. However, it is likely that their size and volume fraction are so small that they are difficult to identify.

Table 1 presents the results of a study of the mechanical properties of the Cu-0.5%Cr-0.08%Zr alloy in various microstructural states. Therefore, the RS of the alloy in both initial states leads to its significant strengthening with a simultaneous decrease in ductility due to a strong refinement of the microstructure. The ultimate tensile strength (UTS) increased from 227 ± 9 MPa to 433 ± 5 MPa after RS with a drop in ductility from 61.0 ± 1.5% to 16.2 ± 0.6% in the case of a quenched alloy. It is important to note that aging leads to additional strengthening of the alloy due to the precipitation of fine particles. The aging of the quenched alloy at 500 °C for 2 hours leads to an increase in the ultimate tensile strength up to 442 ± 19 MPa. In this case, the particle precipitation reduces the ductility of the alloy by almost three times relative to the initial state (to 24.3 ± 3.2%). The aging of the alloy after quenching and RS also leads to an increase in strength characteristics (the UTS of the alloy increases up to 557 ± 18 MPa). However, no decrease in ductility relative to the deformed state of the alloy is observed. During the deformation, two processes simultaneously develop in the alloy: dispersion strengthening and a decrease in the density of defects. Apparently, the course of these two processes acting on the ductility with the opposite sign does not change its values. It should also be noted that the highest degree of deformation achieved in the alloy after extrusion and RS helps to achieve the highest strengthening of the alloy (the UTS of the alloy increases to 597 ± 9 MPa). At the same time, the ductility of the alloy remained at a fairly high level (10.9 ± 0.9%). However, subsequent heating leads to a drop in strength (to 537 ± 10 MPa) with an increase in ductility (to 13.9 ± 1.1%). 

**Figure 6:**
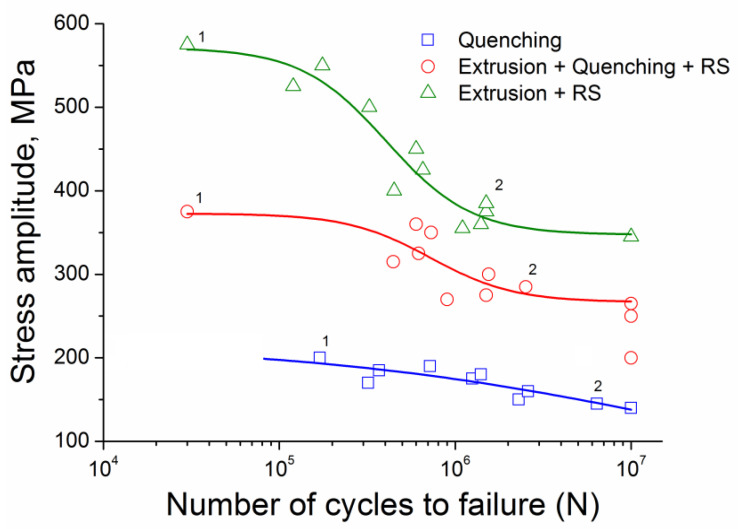
The Woehler (S-N) curves for cyclic deformation of Cu-0.5%Cr-0.08%Zr alloy in different microstructural states.

Products made of low-alloy copper alloys, for example, resistance welding electrodes, operate under cyclic loads. Therefore, the cyclic strength of these materials is also one of their most important functional properties. Cyclic strength was investigated by plotting the fatigue curves of the alloy in various microstructural states in this work. The results of studying the fatigue strength of the Cu-0.5%Cr-0.08%Zr alloy before and after RS are shown in Figure 6. 

The studies have shown that RS leads to an increase in the fatigue strength of the Cu-0.5%Cr-0.08%Zr alloy for both initial states. The fatigue limit of the alloy in the quenched state is 140 MPa. The grain refinement of the quenched alloy caused by RS increases the fatigue limit up to 265 MPa. The highest value of fatigue strength is obtained for the alloy after extrusion and RS, where the fatigue limit is 345 MPa. The combined increase in static and cyclic strength will lead to an increase in the service life of the final product (for example, resistance welding electrode), which in turn will reduce operating costs. 

**Figure 7:**
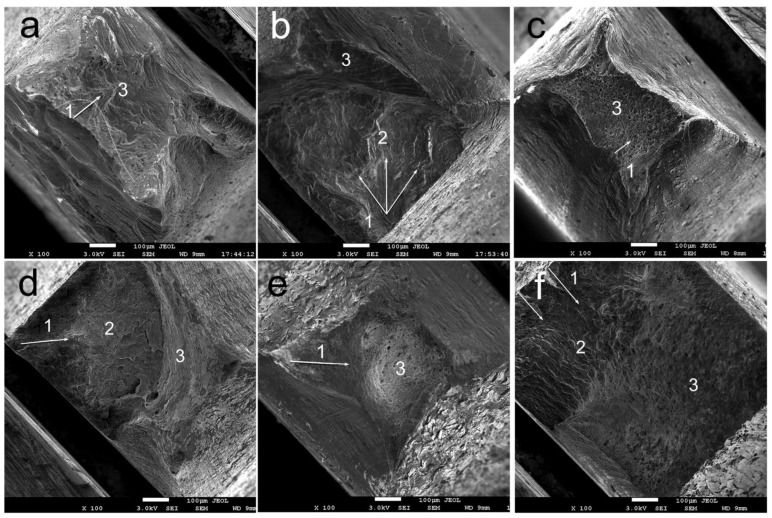
Fracture surfaces of fatigue failure of the Cu-0.5%Cr-0.08%Zr samples after quenching ((**a**) stress is 200 MPa, N = 1.7 × 10^5^; (**b**) stress is 145 MPa, N = 6.4 × 10^6^), extrusion + quenching + RS ((**c**) stress is 375 MPa, N = 3 × 10^4^; (**d**) stress is 275 MPa, N = 1.5 × 10^6^) and extrusion + RS ((**e**) stress is 575 MPa, N = 3 × 10^4^; (**f**) stress is 385 MPa, N = 1.5 × 10^6^). The white arrows indicate the fatigue crack propagation direction.

Figure 7 shows the results of a study of fracture surface fractography after fatigue testing. The studies were carried out for samples that failed in the region of the least number of cycles to failure (point 1 on the Woehler (S-N) curve) and in the region of the largest number of cycles to failure (point 2 on the Woehler (S-N) curve). For samples at point 1, the fracture is characterized by a typical ductile dimple fracture mechanism in all states of the alloy. The zone of initiation and growth of a fatigue crack is weakly expressed. Rapid crack propagation is observed. Pits with a size of about 3–10 µm have an oval shape elongated in the direction of crack growth (Figure 7a,c,d). The type of destruction changes for samples that failed in the area of point 2. Three zones can be distinguished on the fracture surface: the zone of slow initial (threshold) crack propagation (1), the zone of stable crack growth (2) and the static final break zone (3). A rather flat granular relief of the fracture surface characterize the zone of initial crack propagation (1). At the same time, the size of this zone for the quenched alloy is noticeably smaller compared to the deformed alloy, apparently due to the coarse-grained structure. The formation of a distinct corrugated relief with grooves lying across the direction of crack growth is noted in the zone of stable crack growth (2). It should also be noted that the direction of propagation of a fatigue crack in the case of a quenched alloy characterized by a coarse-grained state is different for different grains, as evidenced by the different direction of fatigue grooves (Figure 7b). The relief of the surface of fractures of RS-treated samples differs from the surface relief of quenched samples. The fracture is characterized by greater toughness in the case of the deformed state, probably due to the finer grain. At the same time, it is important to note that delamination and chips are observed on the fracture surface of the alloy samples after quenching and RS, which may indicate the presence of microcracks formed during the RS of the quenched alloy (Figure 7d). The relief of the fracture surface is the most ductile in the case of alloy samples after extrusion and RS, probably due to the presence of particles of the chromium and zirconium phases. It should also be noted that the high strength of the alloy after RS can be the reason for the absence of the stage of accelerated crack development. The final (static) fracture of the sample occurs the moment when the crack reaches the critical length at the stage of its stable growth.

## 4. Discussion

Microstructural changes during extrusion, quenching and rotary swaging lead to a significant increase in the strength of the Cu-Cr-Zr alloy. The difference in the strength characteristics of the alloy swaged in different initial states is about 165 MPa. The subsequent aging leads to level the effect of pre-treatment, and the difference in yield stress and ultimate tensile strength does not exceed 20 MPa. The change in strength (σ) of dispersion-strengthened alloys can be described by the equation [28]:

(1) $$\sigma =\sigma_{0}+\sigma_{\mathrm{part}}+k_{y}D^{-0.5}+\alpha MGb\sqrt{\rho }$$

where *σ*_0_—lattice friction stress, *σ_part_*—particle strengthening, *D*—grain size, *M*—Taylor factor (3.06 for fcc materials), *G*—shear modulus (42.1 GPa for copper [31]), *b*—Burgers vector (2.56 × 10^−10^ m for copper [31]), *ρ*—dislocation density, *k_y_* and *α*—constants.

Particle strengthening in the case of a Cu-Cr-Zr alloy can be estimated using the Orowan equation in the following form [32]:

(2) $$\sigma_{\mathrm{part}}=\frac{0.4MGb\ln(2R/b)}{\pi \lambda \sqrt{1-\nu }}$$

where *R*—particles radius, *λ*—interparticle distance, which can be estimated as *λ* = (3*f*/(2*πrR*^2^))^−0.5^ [33], *ν*—Poisson’s ratio, which is equal to 0.35 for copper [31], *f*—volume fraction of particles.

Since the size of the grain–subgrain structure in alloys weakly depends on pretreatment, the main strengthening mechanisms that determine the difference in mechanical properties can be considered as particle and dislocation strengthening. The data on structural parameters and significant strengthening contributions to the YS are presented in Table 2. It is clearly seen that the high strength of the alloy after extrusion and swaging is determined by the high density of dislocations and the dispersed particles. Aging leads to a slight growth of particles and their volume fraction, the development of recovery and, as a result, a decrease in dislocation strengthening in the extruded alloy. At the same time, the aging promotes the precipitation of finer dispersed particles in the alloy after quenching and RS, which strengthens the alloy and effectively prevents the development of recovery and recrystallization. The predominantly chromium phase precipitates during the aging process, which have good agreement with the literature, should be noted [34]. Cheng et al. found that the order of precipitates in the Cu-0.69Cr-0.10Zr-0.02Mg alloy during aging at 450 °C (8 h) and 550 °C (8 h) changes slightly. The sequence of precipitation after aging at 450 °C was determined as: SSSS → Guinier–Preston zone (face-centered cubic phase) → ordered Cr-rich phase (face-centered cubic) → ordered Cr-rich phase (body-centered cubic). After aging at 550 °C, the sequence of precipitation had small changes concerning the absence of Guinier–Preston zone formation. In this case, the sequence of precipitation was determined as: SSSS → ordered Cr-rich phase (face-centered cubic) → ordered Cr-rich phase (body-centered cubic) [34].

The results obtained in this work show the promise of using RS to gain copper alloys with a high level of strength and electrical conductivity at an acceptable level of ductility. A comparison of the properties of alloys obtained using RS with the properties obtained after other deformation methods also confirms the promise of the method (Table 3). Therefore, Huang et al. obtained similar values of strength (YS = 590 ± 13 MPa, UTS = 612 ± 11 MPa) and electrical conductivity (84.7 ± 1.4% IACS) after the application of RS on the Cu-0.3%Cr-0.1%Zr-0.05Mg alloy; however, the ductility of the alloy is more than two times inferior to the values obtained by the authors of this work (4.6 ± 0.4%) [35]. The strength of low-alloy copper alloys obtained after cold rolling or KOBO extrusion is noticeably less than the values obtained in the present work [3,36]. At the same time, it should be noted that the use of SPD methods leads to a greater strengthening of alloys. For example, the strength of the Cu-0.7wt%Cr-0.07wt%Zr alloy after HPT was 805 MPa [11], and the strength of the Cu-0.44Cr-0.2Zr alloy after equal channel angular pressing (ECAP) reached 700 MPa [37]. However, SPD methods have several disadvantages. First, the workpieces obtained by HPT and ECAP are small, which makes it difficult (and in the case of HPT makes it impossible) to manufacture the final product. Secondly, these methods are still poorly implemented in practice in the industry. Additionally, the application of these methods is noticeably harder compared to RS.

Summarizing the obtained data, it should be noted that RS leads to a significant increase in strength without a dramatical loss of ductility and electrical conductivity. The combination of extrusion and RS increases the strength of the Cu-0.5%Cr-0.08%Zr alloy to ~600 MPa with good ductility (~11%) and electrical conductivity (~82%IACS). This treatment also leads to an increase in the fatigue life of the Cu-0.5%Cr-0.08%Zr alloy up to 345 MPa. Therefore, RS is a promising method for obtaining high-strength low-alloy copper alloys intended for electrical applications.

## 5. Conclusions

1. RS leads to the formation of a grain–subgrain microstructure with both elongated and equiaxed grains and subgrains. In the case of an initially quenched alloy, the subgrain size is ~300–400 nm, and the width of the elongated grains is ~500–600 nm. For the initially extruded alloy, these parameters were ~200 nm and 300–400 nm, respectively. Aging leads to a slight increase in the size of the structural elements for both initial treatments;
2. The aging at 500 °C for 2 h leads to an increase in the microhardness and electrical conductivity of the alloy quenched and swaged after quenching due to the decomposition of SSSS and precipitation of Cr particles with the Nishiyama–Wasserman orientation relationship;
3. Microstructure refinement caused by RS leads to an increase in the UTS of the alloy up to 443 ± 5 MPa and 597 ± 9 MPa with a drop in ductility up to 16.2 ± 0.6% and 10.9 ± 0.9% for the pre-quenched and pre-extruded alloy, respectively. The high strength of the pre-extruded alloy is caused by significant dispersion strengthening (about 130 MPa). In this case, the subsequent aging of the deformed quenched alloy leads to additional strengthening up to 557 ± 18 MPa due to the precipitation of fine Cr particles and a slight drop in UTS to 537 ± 10 MPa in the preliminarily extruded alloy due to the development of recovery;
4. RS increases the fatigue limit of a pre-quenched alloy up to 265 MPa, and that of a pre-extruded alloy up to 345 MPa;
5. The best combination of properties was obtained by combining extrusion and RS, which made it possible to increase the strength of the Cu-0.5%Cr-0.08%Zr alloy up to 597 ± 9 MPa with ductility of 10.9 ± 0.9% and electrical conductivity of 82.0 ± 1.7% IACS.

## Figures and Tables

**Table 1:** Mechanical properties and electrical conductivity (%IACS) of the Cu-0.5%Cr-0.08%Zr alloy in various microstructural states.

Treatment		YS, MPa	UTS, MPa	El, %	%IACS
Quenching	Before aging	72 ± 4	227 ± 9	61.0 ± 1.5	36.2 ± 0.7
	After aging	348 ± 2	442 ± 19	24.3 ± 3.2	81.2 ± 1.6
Extrusion + Quenching + RS	Before aging	426 ± 5	433 ± 5	16.2 ± 0.6	37.9 ± 0.7
	After aging	516 ± 20	557 ± 18	17.1 ± 2.6	83.4 ± 1.8
Extrusion + RS	Before aging	592 ± 6	597 ± 9	10.9 ± 0.9	82.0 ± 1.7
	After aging	504 ± 12	537 ± 10	13.9 ± 1.1	87.0 ± 1.8

**Table 2:** Particle (*σ_part_*) and dislocation (*σ_disl_*) strengthening of the Cu-0.5%Cr-0.08%Zr alloy in various microstructural conditions with the size (*R*) and volume fraction (*f*) of bcc Cr particles, interparticle distance (*λ*) and dislocation density (*ρ*).

Condition		*R*, nm	*f*	*λ*, nm	*σ_part_*, MPa	*Ρ* × 10^14^, m^−2^	*σ_disl_*, MPa	*σ_part_* + *σ_disl_*, MPa
Extrusion + Quenching + RS	Before aging	-	-	-	-	4.9	176	176
	After aging	1.75	0.00066	98.6	129	4.5	168	297
Extrusion + RS	Before aging	1.9	0.00071	103.1	128	5.5	185	313
	After aging	2.4	0.00094	113.2	128	3.6	150	278

**Table 3:** Mechanical properties of Cu-Cr-Zr alloys (E—extrusion; Q—quenching; A—aging; PA—pre-aging; R—rolling; CR—cold rolling; ST—solution treatment; HPT—high pressure torsion; ECAP—equal channel angular pressing; MATE—multi-angular twist channel extrusion).

Alloy	Condition	YS, MPa	UTS, MPa	El, %	% IACS	Reference
Cu-0.5%Cr-0.08%Zr	Q + A (500 °C, 2 h)	348 ± 2	442 ± 19	24.3 ± 3.2	81.2 ± 1.6	Present study
	E + Q + RS + A (500 °C, 2 h)	516 ± 20	557 ± 18	17.1 ± 2.6	83.4 ± 1.8	
	Extrusion + RS	592 ± 6	597 ± 9	10.9 ± 0.9	82.0 ± 1.7	
Cu-0.3%Cr-0.1%Zr-0.05Mg	PA (450 °C, 1 h) + RS + A (375 °C, 1 h)	590 ± 13	612 ± 11	4.6 ± 0.4	84.7 ± 1.4	[35]
Cu-0.4wt%Cr-0.3 wt%Zr	A (450°C, 3 h) + CR (80%)	545 ± 6	568 ± 5	1.5 ± 0.3	75.3 ± 0.2	[3]
Cu-(0.6–0.8)%Cr (0.07–0.09)%Zr	ST + A + wire drawing (ε = 0.89)	-	585.7	9.3	78.7	[8]
Cu-0.7wt%Cr-0.07 wt%Zr	Q + HPT (N = 15) + A (450 °C, 1 h)	785	805	13	~60	[11]
Cu-1Cr-0.1Zr	KOBO extrusion	455	522	18.7	-	[36]
Cu–0.44Cr–0.2Zr	Q + ECAP (Bc, N = 8) + A (425 °C, 1 h)	650 ± 20	700 ± 40	-	-	[37]
Cu–0.44Cr–0.2Zr	Q + ECAP (Bc, N = 4)	554	~600	-	-	[38]
Cu-0.7Cr-0.19Zr	PA (150 °C, 5 h) + A (425 °C, 5 h)	275 ± 4	409 ± 3	15.1 ± 0.5	70.8 ± 0.2	[39]
Cu-1.04%Cr-0.08%Zr	PA (480 °C, 2 h) + R + A (420 °C, 1 h)	547 ± 1	562 ± 2	22.5 ± 1.0	84.1 ± 0.1	[40]
Cu-0.096%Cr-0.07%Zr	ECAE-Conform (N = 8)	~550	~600	~10	-	[41]
Cu-0.6%Cr-0.48%Zr	MATE	440.2	645.1	9.3	-	[42]
Cu-0.8Cr-0.6Zr	ECAP (N = 4) + A (450 °C, 4 h)	-	577.2	-	78.5	[43]

## Data Availability

All the data required to reproduce these experiments are present in the article.
